# Recovery markers in elite climbers after the national boulder climbing championship

**DOI:** 10.3389/fspor.2024.1251047

**Published:** 2024-02-09

**Authors:** Arthur Fernandes Gáspari, Mayni Gabriele Zaminiani, Manoela de Carvalho Vilarinho, Danilo Caruso, Patricia dos Santos Guimarães, Rafael Perlotti Piunti, Alex Itaborahy, Antonio Carlos de Moraes

**Affiliations:** ^1^ School of Physical Education, University of Campinas, Campinas, Brazil; ^2^ Brazilian Sport Climbing Association, São Paulo, Brazil; ^3^ Health Technology Assessment Unit, National Institute of Cardiology, Rio de Janeiro, Brazil

**Keywords:** sport climbing, rock climbing, exercise recovery, muscle damage, exercise readiness

## Abstract

This study aimed to investigate recovery markers among elite climbers following the National Boulder Championship. We assessed maximum isometric hand grip strength (HS), forearm swelling (circumference), delayed soreness in forearm muscles, tiredness, and exercise readiness at several time points: pre-competition, immediately post-competition (within 4 min after their last effort), and 12, 24, 48, and 60 h post-competition. Maximum isometric hand grip strength decreased by 6.38 ± 1.32% (*p* = 0.006) post-12 h, returning to pre-competition values post-24 h (all *p* > 0.05). Forearm circumference (FC) increased 1.78 ± 1.77% (*p* < 0.001) post-competition, returning to pre-competition values post-12 h (all *p* > 0.05). Forearm pain (FP) increased post-competition (*p* = 0.002) and post-12 h (*p* < 0.001), returning to pre-competition values post-24 h (all *p* > 0.05). Tiredness increased post-competition (*p* < 0.001), post-12 h (*p* < 0.001), and post-24 h (*p* < 0.001), returning to pre-competition values post-48 h (all *p* > 0.05). Climbing readiness was reduced post-competition (*p* < 0.001), post-12 h (*p* < 0.001), post-24 h (*p* < 0.001), and post-48 h (*p* = 0.005), only returning to pre-competition values post-60 h (*p* = 0.189). Visual analysis of individual data pointed out a relatively small variability in the HS and FC markers, while FP, tiredness, and readiness exhibited larger individual variations. These findings indicate that different recovery patterns exist for the analyzed markers, suggesting that athletes may require up to 60 h after a competition to fully recover and regain their ability to face new competitive challenges.

## 1 Introduction

Sport climbing made its debut as an Olympic discipline at the 2020/21 Tokyo Olympic Games (1). Similar to other sports that require a combination of strength and power, the physical demands of training and competitive climbing elicit a range of physiological and metabolic responses in the body, with the magnitude influenced by factors such as the activity's duration, intensity, and frequency (2–5). In the acute phase, overexertion can lead to reduced muscle function, structural damage, and inflammation, often accompanied by subsequent pain, fatigue, and a diminished state of readiness for further exercise. These issues can directly impact an athlete's performance during both competitions and training sessions (6–10).

Sport climbing is categorized into three distinct disciplines: speed, lead, and boulder. The boulder discipline, in particular, involves a series of ascent attempts interspersed by brief periods of rest. Climbers attack routes that are typically around 4 meters in height, often set on more challenging surfaces. This discipline demands a succession of high-intensity bursts of effort to overcome obstacles and reach the top of each route (11). These characteristics require high-intensity, powerful movements, frequent changes in direction, and extensive use of the stretching and shortening cycle. These efforts are often associated with the risk of muscle damage and subsequent declines in performance (12–15).

The potential of climbing to elicit acute performance-reducing psychophysiological responses is enhanced by current models of competition. Sport climbing competitors must perform in qualifying, semi-finals, and finals (depending on the number of participants) on consecutive days and sometimes more than once a day (16). This demanding schedule can become even more exhausting in the context of the current world cup circuit, which often features back-to-back competitions on consecutive weekends (17). Such intensive schedules, with minimal time allocated for recovery, can place substantial strain on athletes and should be the focus of attention for coaches and organizations.

Evaluating an athlete's recovery status involves the assessment of both physiological and psychological factors (18). Therefore, it is crucial to employ methods that can comprehensively investigate both dimensions (18). Various studies have focused on strategies aimed at enhancing and accelerating recovery, and these have gained widespread utilization within sports (7, 8, 12, 15, 19). These strategies can be broadly categorized into regeneration strategies, which apply to physiological aspects, and psychological recovery strategies. In the context of this study, the term “recovery” encompasses both physiological and psychological dimensions (18).

An athlete's performance can be significantly impacted by their state of recovery, which involves a combination of physiological factors, including muscle damage, inflammation, redox state, reduction in energy reserves, and nutritional and hydration status (6, 20, 21). Psychological aspects, such as an increased subjective perception of effort, pain, and tiredness, also play a crucial role (6, 20, 21). For climbing athletes, recovery during and after competitions is a pivotal determinant of sustained performance. Therefore, comprehending the dynamics of recovery following competitive events is essential for designing effective recovery strategies to optimize athlete performance in competitions while also mitigating the risk of overreaching, overtraining, and more serious injuries (6, 7, 12, 19). Thus, our study aims to investigate the temporal changes in both physiological and subjective markers of recovery among elite climbers within the 60 h following the National Boulder Championship. Notably, to the best of our knowledge, multi-day temporal changes in recovery markers among climbers following real or simulated competitions have never been investigated. Thus, being aware of the high level of training of the competitors and possible repeated bout effect occurrence decreasing time to recovery (7, 12, 18), we hypothesized that recovery markers would deteriorate until post-24 h, with progressive recovery up to post-60 h.

## 2 Materials and methods

This study was approved by the Ethics Committee of the University of Campinas (CAAE: 52244421.4.0000.5404) and conducted in accordance with the Declaration of Helsinki. The participants provided their written informed consent to participate in this study.

### 2.1 Subjects

The sample consisted of nine climbers from the Brazilian Sport Climbing Team, comprising four men and five women. In this elite group, six of these athletes had also had experience in an international competition. All athletes competed in the Nationals and participated in the week-long evaluation of the Brazilian Sport Climbing Team that took place the week following the championship. The data collections reported in this study were performed during these two events. Sample characteristics are described below (Tables 1, 2).

**Table 1:** Athletes’ characterization.

	Mean	SD	Minimum	Maximum
Climbing experience (years)	13	3	10	19
Age (years)	28	5	18	34
Body mass (kg)	58.5	8.6	45	72.4
Height (cm)	169.4	6.8	157.5	178.1
Arm span (cm)	174.2	9.9	160.5	191.5
Ape index	1.03	0.02	1	1.08
Lean body mass (kg)	50.3	9.1	39.8	64.4
Body fat (%)	13.7	5.9	6.7	22
Circumferences	** **			
Relaxed arm (cm)	28.4	2.9	24	32.4
Contracted arm (cm)	31.0	3.5	26.2	36.5
Relaxed forearm (cm)	26.5	2.8	23	30.8
Forearm contracted (cm)	27.3	2.7	24	31.1
Relaxed arm–forearm index	1.9	0.9	1	3.8
Contracted arm–forearm index	3.7	1.1	2.2	5.4
Skinfolds	** **			
Subscapular ^7f^ (mm)	9	2	6	13
Triceps^7f^ (mm)	10	5	5	20
Biceps (mm)	4	2	2	9
Chest^7f^ (mm)	6	3	3	11
Middle Axillary ^7f^ (mm)	7	2	5	11
Iliac crest^7f^ (mm)	11	4	7	15
Supraspinale (mm)	8	3	5	12
Abdominal^7f^ (mm)	11	4	6	17
Front thigh^7f^ (mm)	15	9	7	31
Medial calf (mm)	7	5	4	19
Sum (7 folds) (mm)	68	25	43	106
Sum (10 folds) (mm)	87	32	57	137.5

SD, standard deviation; 7f, skinfolds used in the sum of 7 folds; arm–forearm index values calculated by the difference between the arm and forearm measurements. Ape index values calculated by dividing the span by the height. The values for body fat percentage and lean body mass were obtained from the bioimpedance results.

**Table 2:** Athletes’ maximum isometric hand grip strength.

	Mean	SD	Minimum	Maximum
Hand grip strength—dominant hand (kgf)	48.6	11.5	38	72
Hand grip strength—dominant hand (kgf.kg^−1^)	0.8	0.1	0.63	1.03
Hand grip strength—non-dominant hand (kgf)	48.1	11.2	37.7	74
Hand grip strength—non-dominant hand (kgf.kg^−1^)	0.8	0.1	0.65	1.04

### 2.2 Study design

All athletes went through two phases of the National Boulder Championship: Qualifiers, climbing five boulders, in which each athlete had 5 min to climb alternated with 5 min of rest; and Finals, approximately 10 h after the Qualifiers, with four boulders, 4 min to climb, and about 20 min of rest. Recovery curve analysis was assessed by dominant maximum isometric hand grip strength (HS), forearm circumference (FC), forearm pain (FP), tiredness, and readiness. These measures were performed in six moments: pre-competition, soon after (post-competition: within 4 min after the last effort), and 12, 24, 48, and 60 h post-competition. On the day of the competition, no intervention/recommendation regarding the practice of physical exercises, nutrient intake, or water was made. For all moments after the competition, the athletes were instructed not to practice exercises for the upper limbs. If they practiced exercises for the lower limbs, it was suggested to do so at low intensity and duration. In addition, they were instructed not to ingest stimulant drinks within 60 h after the competition. Finally, at 60 h post-competition, sample characterization evaluations were carried out. Athletes answered a brief questionnaire about practice time and international competitive experience (World Cup and/or Sport Climbing World Cup) and performed body composition assessments.

### 2.3 Anthropometric and body composition assessment

Total body mass (BM) (kg) was measured using a digital scale and height (cm) using a stadiometer. Arm and forearm circumference, relaxed and contracted, were measured using a Cescorf anthropometric measuring tape. Trunk and limb skinfolds were measured using a Lange model caliper with 1 mm precision [Cambridge Scientific Instruments (USA)] according to the procedures described by the International Society for the Advancement of Kinanthropometry (2011). The body composition assessment [body fat percentage data (%) and lean mass (kg)] was performed in the morning 60 h post-competition. Athletes fasted and emptied their bladders before the measurement was taken using eight contact electrodes for electrical bioimpedance (Tanita InnerScan 50v, Tokyo, Japan).

### 2.4 Maximum isometric hand grip strength

The test of maximum isometric muscle strength of the fingers/hand, called maximum isometric hand grip strength (HS), was collected using a Jamar-type dynamometer (Grip Saehan, Hydraulic Hand Dynamometer, SH5001), with support adjustments at the base of the thumb and middle phalanx of the fingers customized for each athlete. At the beginning of each strength test, the participants performed the preparation of the musculotendinous structures by warm-up (22). The warm-up consisted of 10 submaximal and increasing contractions based on the personal and subjective assessment of their strength capacity, namely, 2× 20%, 2× 40%, 2× 60%, 2× 80%, and 2× 90% (except the one measured right after the competition, in which the athletes were already warmed up). Similar warm-up protocols have already been used by our group (23, 24). Then, 3 min after the warm-up, the participants performed three attempts to obtain maximum isometric hand grip strength, with a 2-minute interval between attempts. At the time of the test, the participants stood up, with the dominant hand holding the dynamometer and the arm extended at the side of the body. The participants were asked to squeeze the dynamometer as hard and as fast as possible, with a total duration of 3 s (controlled by the evaluators through a stopwatch). The tests were conducted by an experienced technical member, and strong verbal encouragement was given throughout the test. The average of the three trials was used for the final analysis. For the recovery temporal change analysis, just the dominant hand was assessed, while for sample characterization, both hands were assessed. Maximum isometric hand grip strength values were presented in absolute units (kgf) and relative to body mass, calculated by dividing the absolute hand grip strength by the athlete's body mass (kgf·kg^−1^).

### 2.5 Forearm circumference

The circumference of the forearm was measured to indirectly evaluate muscle edema. First, the point of the largest circumference of the relaxed forearm was marked with a permanent ink pen. Circumference measurement was performed three consecutive times, and the highest value was used for the final analysis (25).

### 2.6 Delayed-onset muscle soreness

Forearm pain (FP) was evaluated at rest with the use of a visual analog scale (VAS) (26–28). Subjects were instructed to open and close their hand twice and based on the sensation of pain and to mark with a pen on a continuous line (100 mm considered 100%) their perception of pain on a scale from 0 “none” to 10 “a lot of pain!”.

### 2.7 Tiredness and readiness

The tiredness and readiness variables were assessed through self-response to the questions: “How tired are you right now?” and “How ready are you to climb a difficult boulder right now?” Measurements were performed using VAS (26, 27), as performed for FP.

### 2.8 Statistical analyzes

For the analysis of the recovery curve (measurements over time: pre-competition, post-competition, and 12, 24, 48, and 60 h), one-way analysis of variance (ANOVA) for repeated measures was performed. When appropriate, Dunnett's *post hoc* analysis was used to verify which measurement times after the competition were different from the pre-competition time. Data are reported as mean and standard deviation. The software used for the analysis was STATISTICA 6.0 (StatSoft, Inc., Tulsa, OK, USA). The significance level adopted was *p* < 0.05.

## 3 Results

**Figure 1:**
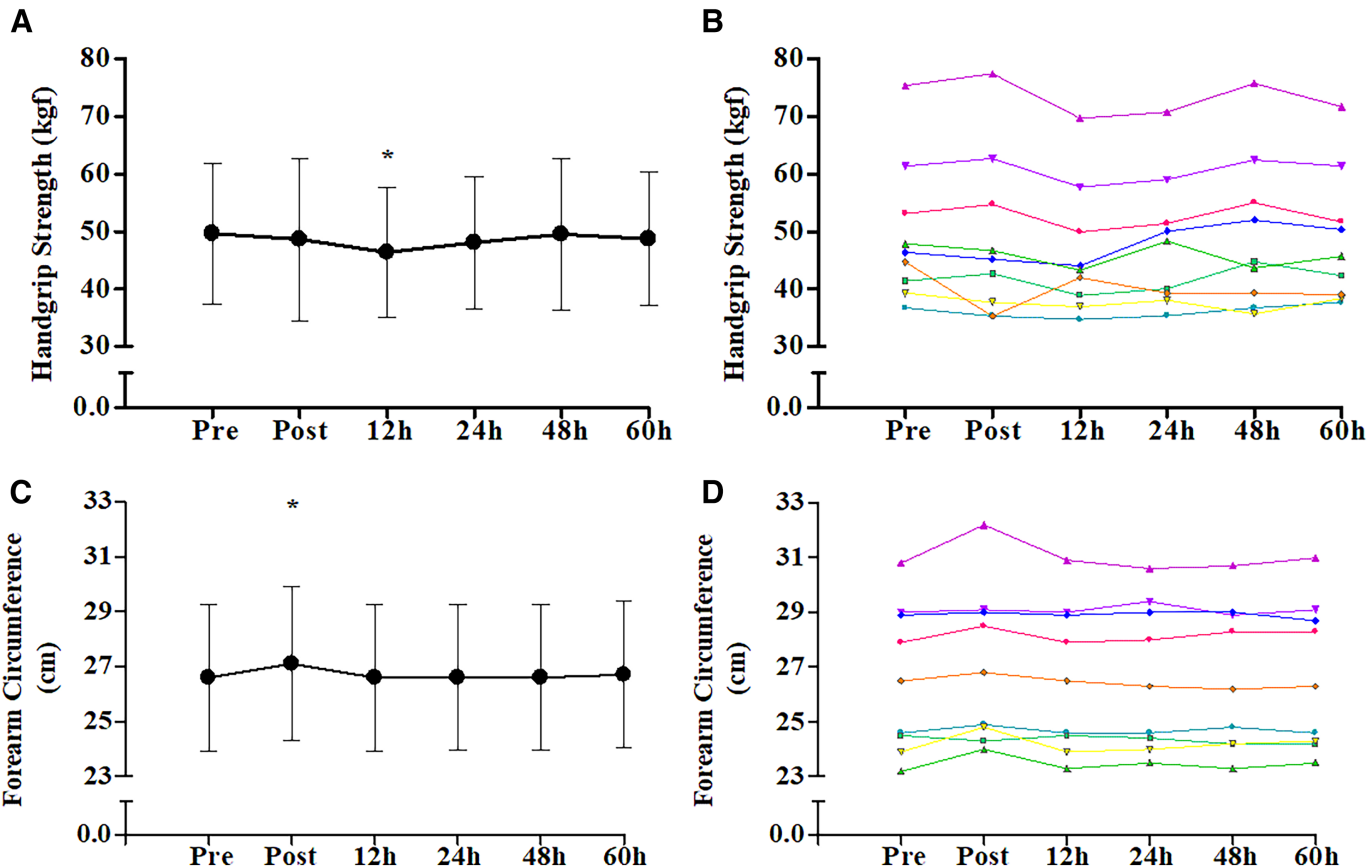
Change through time of physiological markers from pre-competition over 60 h post-competition. (**A**) Maximum isometric hand grip strength mean group values and standard deviation; (**B**) maximum isometric hand grip strength individual values; (**C**) forearm circumference mean group values and standard deviation; (**D**) forearm circumference individual values. *, different compared to pre-competition (Dunnett's *post hoc*; *p* < 0.05).

Figures 1A,B shows the mean and individual values of HS over the six evaluation times. ANOVA showed the main effect of time (*p* = 0.034). Thus, Dunnett's *post hoc* analysis was performed, indicating a reduction of 6.38 ± 1.32% (*p* = 0.006) in HS at post-12 h compared to pre-competition. Meanwhile, the other time changes in HS post-competition (−2.52 ± 7.53%; *p* = 0.452), post-24 h (−2.93 ± 5.32%; *p* = 0.213), post-48 h (−0.43 ± 8.17%; *p* = 0.804), and post-60 h (−1.50 ± 5.98%; *p* = 0.477) were not different from pre-competition. The visual analysis in Figure 1B shows a small variability of individual HS data behavior throughout the 60 h of recovery. Figures 1C,D shows the mean and individual values of the FC over the six evaluation times. ANOVA showed the main effect of time (*p* = 0.001). Thus, Dunnett's *post hoc* analysis was performed, indicating an increase of 1.78 ± 1.77% (*p* < 0.001) in FC post-competition compared to pre-competition. The other time changes in FC post-12 h (0.07 ± 0.12%; *p* = 0.780), post-24 h (0.18 ± 0.66%; *p* = 0.682), post-48 h (0.12 ± 0.89%; *p* = 0.740), and post-60 h (0.37 ± 1.01%; *p* = 0.469) were not different from pre-competition. The visual analysis in Figure 1D shows a small variability of the individual FC data behavior throughout the 60 h of recovery.

**Figure 2:**
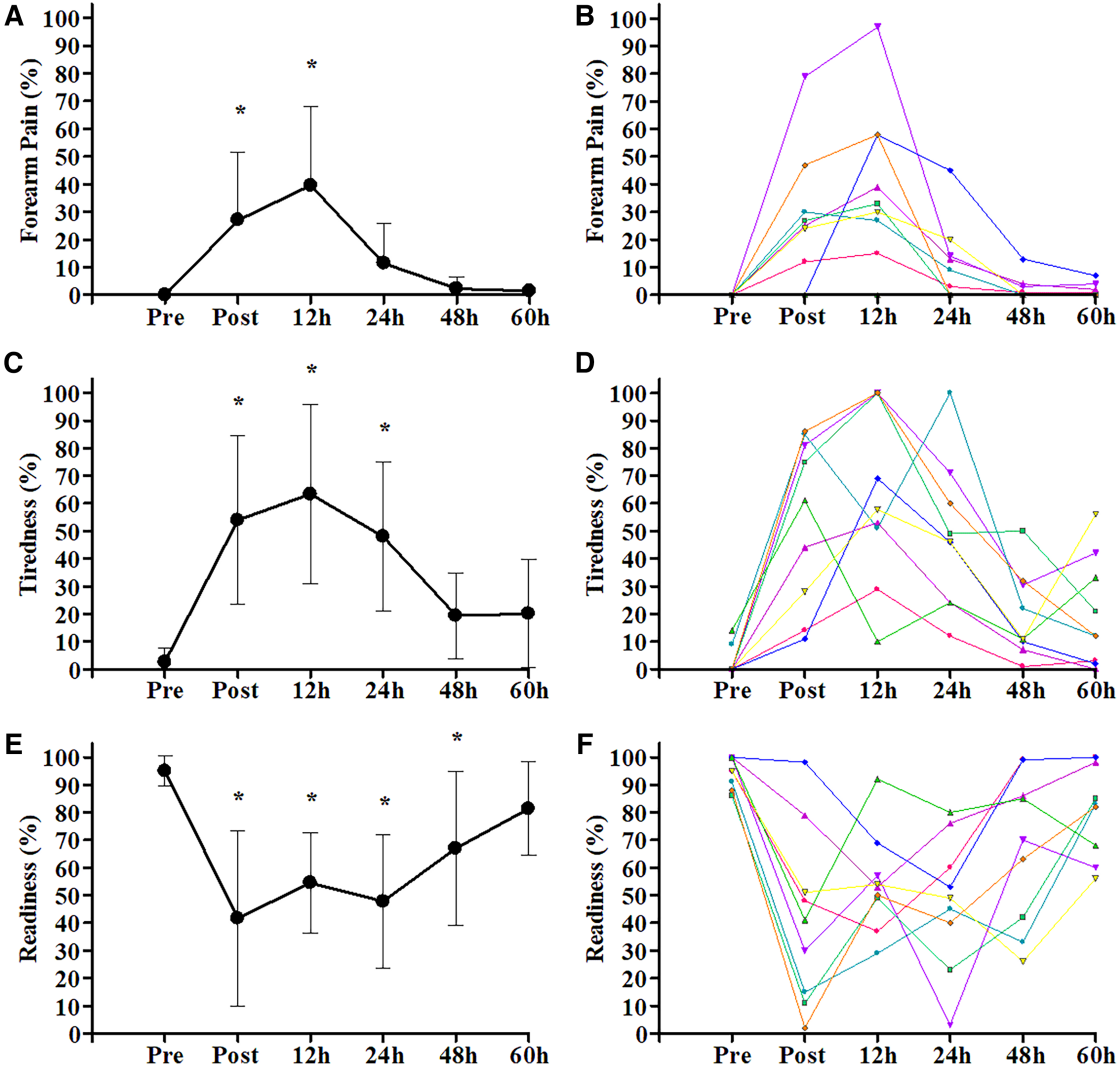
Change through time of subjective perceived markers from pre-competition over the 60 h post-competition. (**A**) Forearm pain mean group values and standard deviation; (**B**) forearm pain individual values; (**C**) tiredness mean group values and standard deviation; (**D**) tiredness individual values; (**E**) readiness mean group values and standard deviation; (**F**) readiness individual values. *, different compared to pre-competition (Dunnett's *post hoc*; *p* < 0.05).

For FP (Figure 2A), ANOVA showed the main effect of time (*p* < 0.001). Therefore, Dunnett's *post hoc* analysis was performed, demonstrating an increase in pain post-competition (*p* = 0.002) and post-12 h (*p* < 0.001) compared to pre-competition. The other time changes in FP post-24 h (*p* = 0.224), post-48 h (*p* = 0.730), and post-60 h (*p* = 0.767) were not different compared to pre-competition. The visual analysis in Figure 2B shows a moderate variability of individual FP data behavior throughout the 60 h of recovery.

Tiredness and readiness for high-intensity bouldering are shown in Figures 2C–F. For tiredness, ANOVA showed the main effect of time (*p* < 0.001), and Dunnett's *post hoc* analysis indicated that post-competition (*p* < 0.001), post-12 h (*p* < 0.001), and post-24 h (*p* < 0.001) were different compared to pre-competition, while post-48 h (*p* = 0.129) and post-60 h (*p* = 0.112) were not different. The climbing readiness ANOVA showed the main effect of time (*p* < 0.001), and Dunnett's *post hoc* analysis indicated that post-competition (*p* < 0.001), post-12 h (*p* < 0.001), post-24 h (*p* < 0.001), and post-48 h (*p* = 0.005) were different from pre-competition, with only the time post-60 h (*p* = 0.189) showing no difference compared to the pre-competition. Finally, the visual analysis in Figures 2D,F shows a large variability of individual tiredness and readiness data behavior throughout the 60 h of recovery.

## 4 Discussion

To the best of our knowledge, this study is the first to investigate recovery markers in elite climbers following a competition. The results reveal that, following a national-level bouldering competition, athletes exhibited a recovery in HS and FP levels within 24 h after the competitive stimulus. In contrast, sensations of tiredness and readiness returned to pre-competition levels at 48 h and 60 h, respectively. These findings, to some extent, deviate from our initial hypothesis. While recovery markers ultimately returned to values similar to the pre-competition state by the 60-h mark, the quickness of the physiological and FP recovery contrasts with our initial expectations. This rapid recovery emphasizes the high training proficiency of the tested athletes and their familiarity with the types of movements encountered in competition. Moreover, it is noteworthy that physiological markers and subjective perceptions did not exhibit a uniform temporal pattern of recovery.

The boulder discipline necessitates brief and intense bursts of effort with limited recovery time between each attempt (29, 30). This highlights the importance of strength and the ability to generate rapid force, particularly from the hands (hand grip), as pivotal factors influencing a climber's performance (31, 32). These types of high-intensity intermittent stimuli, characterized as voluntary and high-magnitude muscle actions, are capable of leading to a reduction in functional muscle capacity, such as in force production, due to fatigue arising from the physiological response to maximal and submaximal voluntary contractions (14, 18, 20) and/or exercise-induced muscle damage (EIMD) (7, 12, 13). EIMD leading to strength loss is attributed to so-called half-sarcomere non-uniformity, which states that the weakest half-sarcomeres accommodate the majority of finer length adjustment, which becomes weaker as muscle lengthening progresses and advances beyond the point of myofilament overlap, and eventually, microtears develop (22, 33). Repeated stretching increases damaged sarcomeres and exacerbates muscle fiber injury, resulting in membrane breakdown and perforation of mechanically activated channels (22, 33). Damage to junctophilins, proteins that connect t-tubules to the sarcoplasmic reticulum membrane and mediate communication between the calcium release channel and the dihydropyridine receptor, may also contribute to strength losses due to excitation–contraction uncoupling (14, 22, 33). This series of events disrupts the excitation–contraction coupling mechanism and the calcium kinetics originating from the sarcoplasmic reticulum, resulting in a decrease in strength (7, 12, 13, 33). Considering the mechanisms elucidated above, our initial expectations were for a substantial decline in HS, coupled with an increase in FC and FP, persisting for a minimum of 24 h following the competition.

We observed a significant difference in HS 12 h post-competition, returning to the pre-competition value after 24 h. This temporal data behavior shows a small individual variability increasing the confidence that the athletes had their HS recovered post-24 h. Notably, an experimental session comprising three repetitions of maximal climbing efforts, with a 2-minute interval of active recovery between each, demonstrated a reduction in HS (32). Heyman et al. (9) also identified a similar decline in strength after a series of repetitions until voluntary exhaustion in “top rope” climbing. The delayed reduction in strength, occurring post-12 h after the stimulus, may indicate that, in addition to competition-induced fatigue, athletes may experience EIMD. This EIMD can trigger an inflammatory response characterized by leukocyte activation, muscle edema, degradation of muscle function, delayed-onset muscle soreness, increased release of muscle proteins into the interstitial space, elevated circulation, and an increase in muscle temperature. The effects of EIMD and the associated muscle soreness, including a decline in muscle strength, may persist for a duration of 12–72 h post-exertion (7, 12, 13, 33).

Peake et al. (13) conducted a literature review, which revealed that EIMD, leading to swelling and diminished strength, tends to peak between 24 and 72 h following the stimulus. Paulsen et al. (34) established a classification system considering low or negligible muscle damage when the decline in force-generating capacity is less than 20%. Consequently, the relatively minor decrease observed in HS and FP values after 24 h, and FC after 12 h, may likely stem from mechanisms in addition to muscle damage. For instance, the increase in FC after a series of climbing bouts can be attributed to the repetitive isometric contractions of the forearm, leading to a reduction in veins blood flow and an increase in forearm swelling. This, in turn, results in a decrease in strength output as swelling and discomfort intensify when the same muscles are contracted and held repeatedly (35).

The values of FC, an indirect indicator of forearm volume and muscle swelling (Figures 1C,D), indicate a significant increase after the competition, returning to pre-competition values post-12 h. Once again, the temporal data behavior shows a small individual variability, increasing the confidence that the athletes’ FC recovery was homogeneous. It is known that strenuous exercise can induce muscle swelling immediately after the stimulus (35), and the persistence of this altered volume accompanied by an inflammatory response is indicative of muscle damage (22, 29, 36–38). Previous studies showed that VO_2_ peak climbing exceeded the VO_2_ peak obtained on an arm ergometer by 102.2%–108.1%, the peak heart rate achieved varied from 162 to 181 bpm, and lactate concentration ranged between 2.4 and 3.9 mmol·l^−1^ after an effort time ranging from 37.2 to 38.6 s, depending on the technical difficulty of the climbing bout (4). We believe that the changes in FC shown in our study are related to the “muscle pump”, a temporary condition that generates muscle swelling described in response to resistance exercise (39). According to Schoenfeld and Contreras (39), high-intensity muscle contractions cause an imbalance in blood supply and drainage in the exercised region. That is due to compression of the veins and preserved normal diameter of the arteries (compression-resistant vascular structure), also leading to an imbalance in the concentration of intramuscular and extramuscular fluids, causing the greatest amount of fluid to be found in the intramuscular space, leading to swelling. This phenomenon has already been described within the practice of climbing (40, 41).

We found that forearm pain experienced a notable reduction within the initial 24 h post-competition. While the individual variability in FP was somewhat greater than that observed for physiological markers, it remained moderate. Nonetheless, the temporal behavior of FP exhibited a similar pattern across individuals, suggesting that although the degree of pain experienced varied among them, the majority demonstrated a significant reduction after the first 24 h. Given that delayed-onset muscle soreness, resulting from muscle damage, typically reaches its peak between 48 and 72 h following the stimulus (42) and that FP did not follow the same temporal pattern as HS, it is plausible that the forearm pain is more likely attributed to minor muscle damages and the breakdown of non-contractile muscle structures rather than substantial muscle damage. Previous studies have highlighted that the fascia is more sensitive than muscle following eccentric contractions (43), which could explain the association between pain and reduced performance, as observed in this study and others (40). A possible explanation for the reduction in force production could be the mechanism of muscle fatigue (44). This phenomenon can manifest at both peripheral and central levels (45). Peripheral fatigue involves intramuscular changes in biochemistry and neuromuscular junctions, while central fatigue is characterized by a decline in neural impulse transmission from the central nervous system to the muscle (43, 45–47). In addition to impairing the ability to generate force, fatigue can be accompanied by sensations of tiredness and exhaustion (45), ultimately constraining high-intensity performance (44, 45).

Repeated muscle contractions can result in the diminished ability to generate or sustain muscle function, a phenomenon commonly referred to as muscle fatigue (48). Fatigue is widely recognized as a critical factor influencing athletic performance, and it comprises a complex event, often characterized by a set of interacting conditions with varying degrees of influence, dependent on the nature of the physical exercise (20, 49). Extensive research has been dedicated to unraveling the fatigue process and its ramifications on performance in physical activities and sports. Nevertheless, a consensus regarding the precise mechanism underpinning this process remains elusive (41–43, 50). In addition to physiological changes, it is vital to pay attention to an athlete's perception of tiredness as it serves as a valuable indicator of their condition (21). Notably, subjective measures capable of gauging tiredness and readiness are typically acquired through verbal feedback and/or the application of specific scales (51, 52). Unlike pain, which often has a precise location, sensations of tiredness, fatigue, and readiness encompass a broader perception of the athlete's overall physical state and their perceived capacity to perform. This subjective dimension adds a layer of subjectivity to the assessment (50–52). Nonetheless, existing literature corroborates that subjective measures are effective in capturing changes in an athlete's well-being as they tend to exhibit greater responsiveness than objective measurements (52). Subjective assessments of athlete recovery, in general, are characterized by their sensitivity and practicality, making them a pivotal component of the recovery–fatigue monitoring process (51).

Unlike FP, the subjective variables, tiredness and readiness (Figures 2C–F), exhibited distinct patterns in this context. Tiredness demonstrated a return to baseline levels around 48 h after the competition, while readiness only reached its initial levels 60 h post-competition. This suggests that athletes might not fully recover from the competitive stimulus when strength and forearm pain recover. These subjective attributes of tiredness and readiness likely account not only for the temporal differences in recovery but also for the considerable variability observed among individuals, emphasizing the importance of individualized assessments and strategies in addressing these aspects. The results obtained for tiredness and readiness led us to believe that they are not entirely linked to local muscle fatigue, given that the forearm has a small muscle mass in relation to the whole. Montull et al. (49) presented a new subjective approach considering that sports performance depends on the athlete's experience and their interactions with the environment. Furthermore, the authors believed that the impairment of these variables may be related to several psychological factors. The studied athletes required at least 60 h of recovery after a competitive stimulus to be fully capable of performing at their maximum performance. The difference in the temporal recovery behavior between physiological markers and subjective markers suggests that for our study there is no strong direct relationship between them, which emphasizes the importance of a holistic understanding of the athlete and the sport, integrating physiological and psychological aspects, considering that both physical and mental factors can influence the athlete's well-being and performance capacity.

This study is not without its limitations. While maximum isometric handgrip strength is recognized as a crucial factor in sport climbing performance, there are other variables, such as rate of force development, finger resistance, and measures related to pulling movements, that could significantly enhance the understanding of the recovery profile of climbers. It is important to note that the maximum isometric handgrip strength measurements were made with a hand dynamometer, and an even more specific strength measurement, such as an instrumented climbing hold, could offer valuable insights. Moreover, it would be intriguing to explore the recovery curve of the non-dominant limb since the intensity imposed on each side can vary non-uniformly based on the characteristics of the climbing routes. In this regard, future research could explore additional variables and employ even more specialized instruments than those used in this study. Furthermore, investigating the temporal changes in recovery variables in other climbing disciplines such as LEAD and SPEED, and following multiple consecutive days of competition, would be of interest and could provide further insights into the recovery requirements in sport climbing.

This study is the first to show the temporal changes in physiological and subjective perceived markers of recovery among elite climbers following a competition. Considering the demanding competition schedules that elite climbers typically face, the findings from this study assume great significance. They offer valuable insights into the evolving recovery patterns of variables pertinent to the preparation and performance of professional sport climbing competitions. This, in turn, can contribute to the mitigation of injury risks arising from physiological and/or psychological stress and potentially prompt a reconsideration of the competition calendar. From a practical standpoint, while physiological markers appear to recover after 24 hours post-competition, allowing athletes to restart physicaloriented training during this period, the more extended temporal patterns and the substantial individual variability observed in subjective markers imply that athletes may require up to 60 hours of recovery to regain their sense of fitness and readiness for competitive endeavors.

## Data Availability

The original contributions presented in the study are included in the article/Supplementary Material, further inquiries can be directed to the corresponding authors.
